# Design, synthesis, and biological evaluation of the novel glycyrrhetinic acid-cinnamoyl hybrids as anti-tumor agents

**DOI:** 10.1186/s13065-016-0222-8

**Published:** 2016-12-01

**Authors:** Wenbo Guo, Mengmeng Yan, Bing Xu, Fuhao Chu, Wei Wang, Chenze Zhang, Xiaohui Jia, Yaotian Han, Hongjun Xiang, Yuzhong Zhang, Penglong Wang, Haimin Lei

**Affiliations:** 1 School of Chinese Pharmacy, Beijing University of Chinese Medicine, No.6 Wangjing Middle Ring South Road, Beijing, Chaoyang District China; 2 Department of Pathology, Beijing University of Chinese Medicine, No.11 North Third Ring Road, Beijing, Chaoyang District China

**Keywords:** Glycyrrhetinic acid, Cinnamic acid, Synthesis, Biological evaluation, Anti-tumor, Combination principle

## Abstract

**Background:**

Glycyrrhetinic acid (GA) derivatives had shown not only cytotoxicity but also could trigger apoptosis in various human cancer cell lines. Moreover, cinnamic acid (CA) and its phenolic analogues as potent antitumor agents were employed in the design of anti-tumor drugs. To further improve the anti-tumor activity of GA and CA derivatives, a series of novel compounds were designed and synthesized using GA and CA derivatives fragments.

**Results:**

The result showed that all the novel glycyrrhetinic acid-cinnamoyl (GA–CA) hybrids presented higher antitumor activity on the tumor cell lines of HepG2, HT-29, Hela and lower cytotoxicity on three non-tumor cells lines MDCK, HY926, H9C2 than the parent compounds (IC_50_ > 50 μM). It was worth noting that **8a** had a superior cytotoxicity effect on Hela cells (IC_50_ = 8.54 μM) than on other cancer cell lines (IC_50_ > 15 μM). And it also indicated that **8a** showed lower cytotoxicity (IC_50_ > 27 μM) towards MDCK, HY926 and H9C2 cells than cisplatin (DDP, IC_50_ < 10 μM). Moreover, according to the acute toxicity, it could be indicated that the LD_50_ of **8a** exceeded 3.0 g/kg by oral administration in mice. The further research using Giemsa, H33342 staining, flow cytometric analysis and caspase-3 assay showed that **8a** could cause Hela cell damage, nuclei lysis and apoptosis. In addition, the structure–activity relationships of these hybrids were briefly discussed.

**Conclusions:**

Compared with GA, target compounds demonstrated better anti-tumor activity, among which **8a** was the most active one. What’s more, structure–activity relationship analysis also revealed that hybrids with *trans* olefinic bond group show higher antitumor activity than those without olefinic bond, such as **1a** **>** **1b**, **6a** **>** **2b**, **8a** **>** **3b**, **9a** **>** **4b**. In addition, it was found that the methoxy substituent might enhance selectivity of GA–CA hybrids towards regular non-cancerous cells MDCK, HY926 and H9C2, such as **4a**, **6a**, **7a**, **8a**. However, there might be less relationship between the cytotoxicity and the quantity, position of methoxy moiety. Hence, it is urgent need to synthesize efficient, low toxicity and multi-target anti-tumor compounds based on the structure combination principle.

## Background

In the process of drug discovery and development, natural products play a highly significant role [1, 2]. A series of pentacyclic triterpenoids were proved to have potent antitumor activity [3–6]. Structural modification of bioactive natural products according to combination principle was an important approach in search for new lead compounds [7–9]. Based on this principle, previous studies in our laboratory had already obtained a series of novel antitumor compounds with promising cytotoxicity [10–12]. Given the potent cytotoxicity and apoptosis-inducing activity of the natural pentacyclic triterpenoid GA, it is becoming a valuable lead compound in the design of anti-tumor drug [13]. It also had been reported that GA possessed selective toxicity to varieties of tumor cells [14, 15]. The previous researches showed the introduction of ester-joined groups at 3-OH of GA could enhance the antitumor effect [16, 17]. Meanwhile, CA and its phenolic analogues were also employed as the active scaffold in the design of anti-tumor drugs for their potent cytotoxicity [13, 18–21]. Moreover, CA moiety could induce selective cytotoxicity in developing anti-tumor agents [21]. To further improve the anti-tumor effect of GA and CA derivatives and find a series of efficient, low toxicity, multi-target GA–CA hybrids, we integrated the GA and CA derivatives fragments into one molecule via an ester bond based on structural combination principle.

## Results and discussion

### Chemistry

**Scheme 1:**
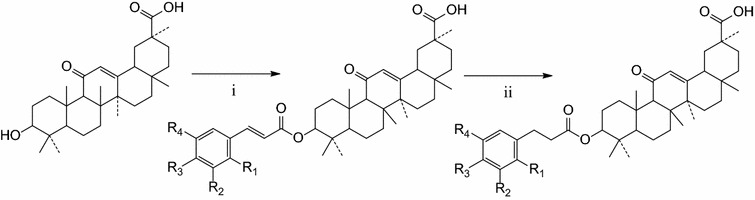
Reagents and conditions: (i) anhydrous CH_2_Cl_2_, DMAP/DCC, 24 h; (ii) THF, Pd/C, H_2_, 2 h

All the designed hybrids were synthesized according to Scheme 1. The coupling reactions between GA and the corresponding CA derivatives were performed using dimethylaminopyridine (DMAP) and dicyclohexylcarbodiimide (DCC) as the catalyst in anhydrous dichloromethane (DCM), to afford glycyrrhetinic acid hybrids **1a–9a**, as shown in Scheme 1. Subsequently, compounds **1a, 6a, 8a, 9a** were further hydrogenated by Pd/C to obtain the target compounds **1b–4b**. The structures of all target compounds (Tables 1, 2) were determined by ^1^H-NMR, ^13^C-NMR and mass spectrometer (ESI–MS).

**Table 1:** The structures of glycyrrhetinic acid hybrids **1a–9a**

No.	R_1_	R_2_	R_3_	R_4_
1a	H	H	H	H
2a	H	H	CH_3_	H
3a	OCH_3_	H	H	H
4a	H	OCH_3_	H	H
5a	H	H	OCH_3_	H
6a	OCH_3_	OCH_3_	H	H
7a	OCH_3_	H	H	OCH_3_
8a	OCH_3_	OCH_3_	OCH_3_	H
9a	H	OCH_3_	OCH_3_	OCH_3_

**Table 2:** The structures of glycyrrhetinic acid hybrids **1b–4b**

No.	R_1_	R_2_	R_3_	R_4_
1b	H	H	H	H
2b	OCH_3_	OCH_3_	H	H
3b	OCH_3_	OCH_3_	OCH_3_	H
4b	H	OCH_3_	OCH_3_	OCH_3_

### Biological activity

#### Cytotoxicity assay

As shown in Table 3, all the synthesized compounds were tested for their cytotoxicity on three tumor cell lines (HepG2, HT-29, and Hela) and three non-tumor cell lines (MDCK HY926 and H9C2) using the standard MTT assay and IC_50_ values for different cell lines were outlined.

**Table 3:** Anti-proliferative effects of glycyrrhetinic acid hybrids

Compound	IC_50_ (μM)^a^					
	Hela	HepG2	HT-29	MDCK	HY926	H9C2
**1a**	23.67 ± 2.98	24.40 ± 1.39	22.34 ± 1.75	22.63 ± 1.38	28.93 ± 1.85	46.13 ± 2.01
**2a**	14.19 ± 1.10	22.08 ± 1.18	21.72 ± 1.81	37.95 ± 1.81	27.63 ± 1.84	>50
**3a**	24.56 ± 1.72	31.99 ± 1.90	22.16 ± 2.34	22.93 ± 2.43	>50	>50
**4a**	17.53 ± 1.52	12.67 ± 1.10	14.88 ± 1.80	22.24 ± 2.56	31.01 ± 1.87	>50
**5a**	23.38 ± 1.38	31.15 ± 1.34	32.99 ± 1.92	21.67 ± 1.01	>50	36.14 ± 1.92
**6a**	22.48 ± 2.08	24.36 ± 1.44	24.39 ± 1.76	33.67 ± 1.40	24.99 ± 1.38	>50
**7a**	28.01 ± 1.63	25.41 ± 1.97	26.41 ± 1.96	>50	45.07 ± 2.25	>50
**8a**	8.54 ± 1.44	21.47 ± 1.50	15.02 ± 1.26	31.84 ± 2.79	27.73 ± 1.39	>50
**9a**	30.67 ± 1.89	21.88 ± 1.41	25.52 ± 1.29	27.72 ± 1.08	30.79 ± 1.37	47.43 ± 1.07
**1b**	26.92 ± 1.74	32.29 ± 1.00	28.63 ± 2.99	33.33 ± 2.98	33.65 ± 3.09	>50
**2b**	23.13 ± 2.33	33.08 ± 2.09	29.34 ± 2.22	41.30 ± 1.48	>50	45.75 ± 1.80
**3b**	22.54 ± 2.16	27.31 ± 1.95	26.21 ± 1.24	>30	38.46 ± 2.02	>50
**4b**	28.69 ± 1.68	22.38 ± 1.29	22.35 ± 1.2	>30	>50	>50
**GA**	>50	>50	>50	>100	>100	>100
**DPP**	3.76 ± 0.38	4.57 ± 0.85	5.28 ± 0.74	9.97 ± 1.12	5.12 ± 0.71	5.31 ± 0.26

^a^IC50 values were calculated using GraphPad Prism 5.01. Data were shown as mean ± SD (n = 3) from three independent experiments

After combination, most of the synthesized compounds showed improved cytotoxicity compared to GA. Among them, compound **8a** demonstrated better cytotoxicity (IC_50_ = 8.54 μM) against Hela. Structure–activity relationship analysis among **1a**, **6a**, **8a**, **9a**, **1b–4b** also revealed that compounds with *trans* olefinic bond group seemed to more active than those without olefinic bond, such as **1a** > **1b**, **6a** > **2b**, **8a** > **3b**, **9a** > **4b**. This structure–activity relationship analysis was in agreement with our previous study in designing neuroprotective agents [9]. Compared to **1a** (IC_50_ < 25 μM), it was found that the methoxy substituent might enhance cytotoxicity selectivity of GA–CA hybrids towards tumor cell lines, such as **4a**, **6a**, **7a**, **8a**. This was in accordance with the previous study that dihydroartemisinin-cinnamic acid ester hybrids with methoxy moiety displayed highly selective cytotoxicity against the human lung carcinoma A549 cells, although it showed low cytotoxicity on non-tumor hepatic L-02 cells [21]. However, there might be less relationship between cytotoxicity and the quantity, position of methoxy moiety. These findings may provide a new framework for the design of new GA hybrids as anti-tumor drugs.

#### Cytotoxicity selectivity

**Fig. 1:**
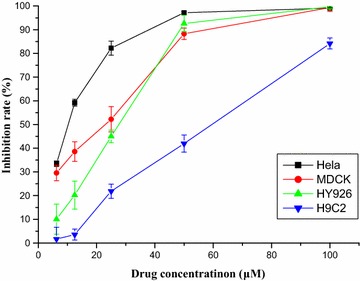
The different inhibition activity of **8a** for Hela, HY926, MDCK and H9C2 cells

Based on the above evidence, **8a** showed selective cytotoxicity towards Hela cell lines. The different inhibition activity of **8a** for Hela, HY926, MDCK and H9C2 cells are shown in Fig. 1 detailedly. Taken together, the results demonstrated that **8a** displayed not only selective cytotoxicity on Hela cell lines, but also a concentration-dependent manner.

From the obtained results, we can see the inhibition rate of **8a** at 12.5 μM against MDCK, HY926, H9C2 and Hela were 38.56, 20.20, 3.56 and 59.20%. Hence, in a certain degree, compound **8a** might have cytotoxicity selectively towards Hela.

#### Acute toxicity

As the above results showed, **8a** had a selective cytotoxicity against tumor cells. In order to test its safety, the acute toxicity of **8a** was evaluated by gavage. During the 2 weeks after oral administration of the maximum tolerated dose (3 g/kg), no signs of toxicity or deaths were observed.

#### Giemsa staining on Hela cells

**Fig. 2:**
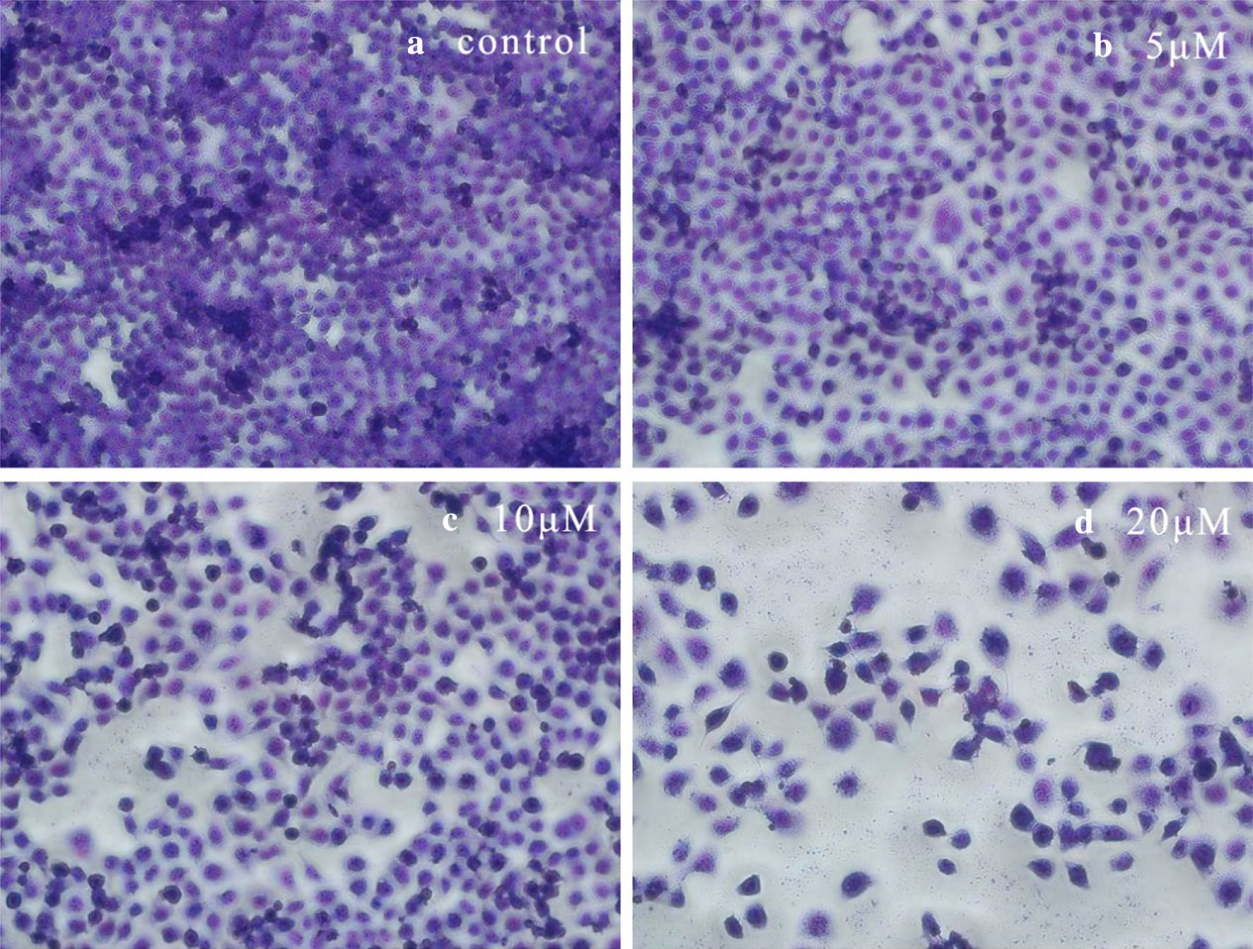
Morphological changes observation of Hela cells by Giemsa staining (×200): **a** control group without **8a**; **b** 5 μM of **8a**; **c** 10 μM of **8a**; **d** 20 μM of **8a**. The cell morphology was observed and photographed under inverted phase-contrast microscope after Giemsa staining

To confirm whether the apoptotic morphological changes could be associated with **8a**, Hela cells were treated with various concentrations of **8a** for 72 h and then used Giemsa staining. The morphology changes were observed and photographed under inverted phase-contrast microscope at a magnification of 200×. With the increase of drug concentration, the process of cell loss, nuclei lysis, chromatin condensation and cytoplasmic shrinkage were aggravated (Fig. 2).

#### H33342 staining on Hela cells

**Fig. 3:**
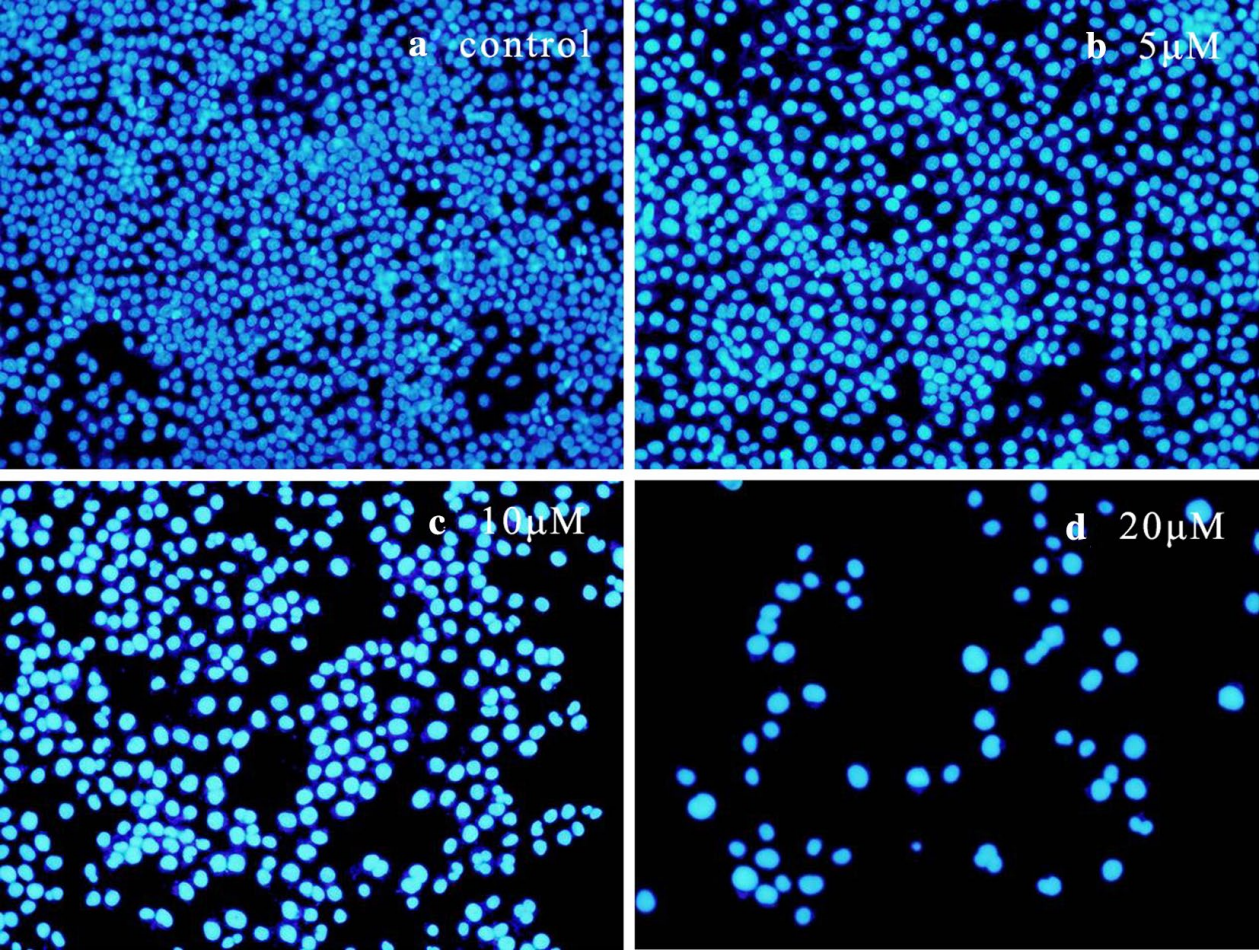
Morphological changes observation of Hela cells by H33342 staining (×200): **a** control group without **8a**; **b** 5 μM of **8a**; **c** 10 μM of **8a**; **d** 20 μM of **8a**. The cell morphology was observed and photographed under inverted phase-contrast microscope after H33342 staining

To confirm whether the characteristic nuclear changes could be associated with **8a**, Hela cells were treated with various concentrations of **8a** for 72 h and then used H33342 staining. The morphology changes were observed and photographed under inverted phase-contrast microscope at a magnification of 200×. With the increase of drug concentration, the nuclear fragmentation, the cytoplasmic shrinkage, and the shape of apoptotic cells became irregular (Fig. 3).

#### Annexin V-FITC/propidium iodide (PI) assay

**Fig. 4:**
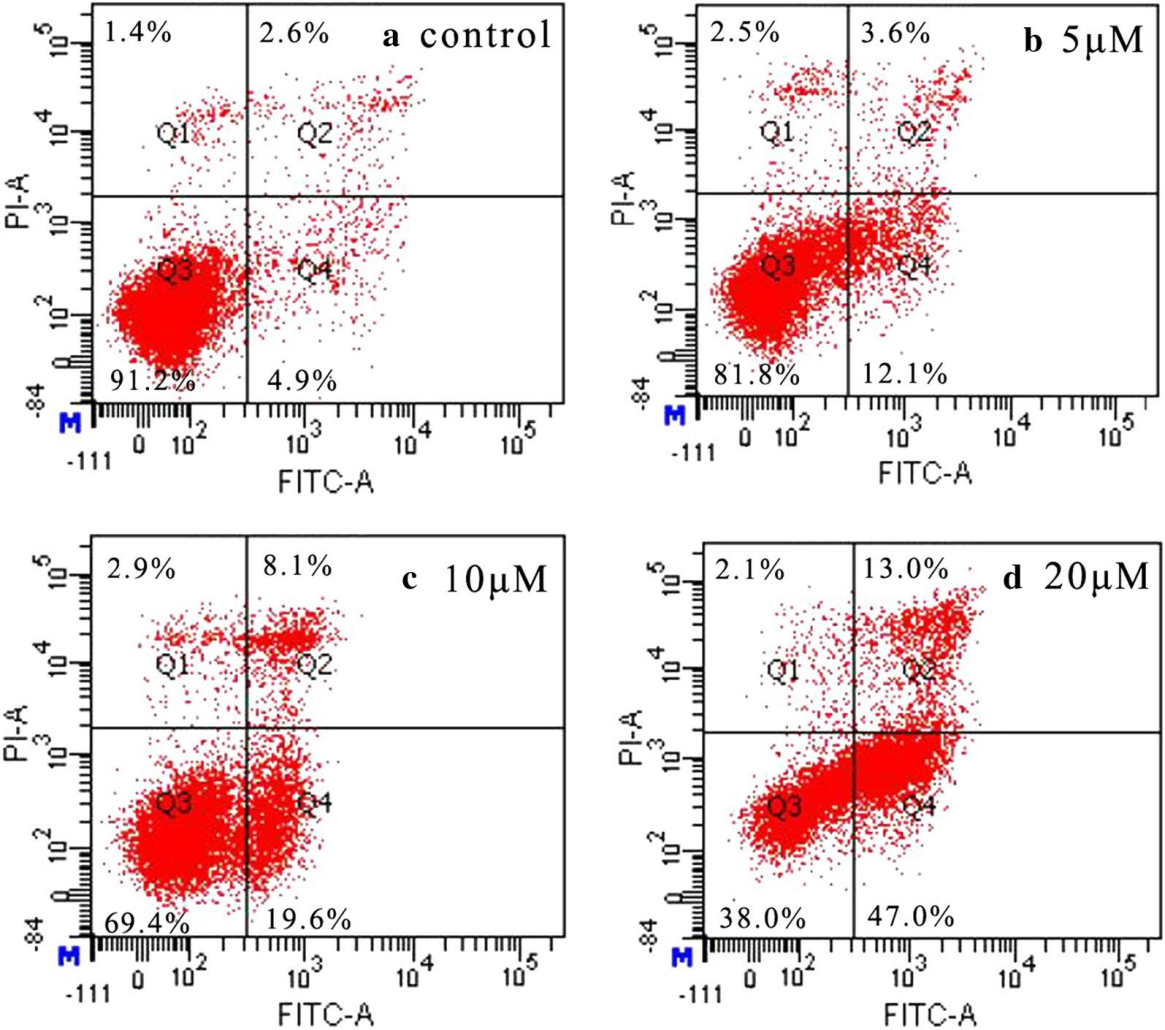
Apoptosis analysis by FCM using AnnexinV-FITC/PI staining on the Hela cells treated by **8a**; **a** control group without **8a**; **b** 5 μM of **8a**; **c** 10 μM of **8a**; **d** 20 μM of **8a**

The effects of **8a** on apoptosis in Hela cells were further determined by flow cytometric analysis. Cells were treated with **8a** at three concentrations of 5, 10, 20 μM and then stained with both annexin V-FITC and PI. The flow cytometry observed four quadrant images: the Q1 area represented necrotic cells, the Q2 area represented late apoptotic cells, the Q3 area represented intact cells and the Q4 area represented the early apoptotic cells. The results were shown in Fig. 4. The apoptosis ratios of **8a** were found from 15.7% (5 μM) to 27.7% (10 μM) and 60% (20 μM) which increased gradually in a concentration manner, respectively, while that of the control was 7.5%. It was indicated that **8a** was able to significantly induce Hela cells apoptosis. This was in accordance with previous reports that GA and CA hybrids could induce cancer cell apoptosis [5, 20].

#### Caspase-3 assay

**Fig. 5:**
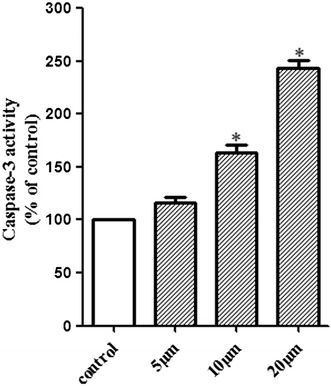
Caspase-3 activity of **8a** on Hela cells. Values of caspase-3 activity are reported as mean ± SD (n = 3). *Compared with control group, *P* < 0.05

From the results of flow cytometry analysis, it could be clarified that **8a** was able to significantly induce Hela cells apoptosis. What’s more, caspase-3 plays a crucial role in the process of apoptosis induced. The method of measuring the levels of ρ-nitroanilide cleaved from the substrate N-Ac-DEVD-ρNA was followed to determine the caspase-3 activity. As shown in Fig. 5, caspase-3 activities in Hela cells were enhanced in a concentration-dependent manner after the cells were exposed to **8a**, from which it could be implicated that caspase-3 was activated by **8a** to promote the apoptosis of the cells.

## Conclusions

Studies on synthetic glycyrrhetinic acid derivatives and their bioactivities drew considerable attention in the past several years. Our experimental findings suggested that all GA–CA hybrids showed better cytotoxicity than the parent materials in tested cancer cells. Among the active compounds, **8a** presented a superior cytotoxicity effect on Hela cells (IC_50_ = 8.54 μM) than on other cancer cell lines and exhibited lower cytotoxicity towards three non-tumor cells lines (MDCK, HY926, H9C2) than DDP. Furthermore, structure–activity relationship analysis revealed that hybrids with *trans* olefinic bond group were more active than those without olefinic bond, and we found that the methoxy substituent could enhance cytotoxicity selectivity of GA–CA hybrids. In order to further confirm our conclusion, we combined Giemsa, H33342 staining, flow cytometric analysis and caspase-3 assay indicated that **8a** could induce Hela cells damage, nuclei lysis, block intercellular contact and apoptosis. From these results, it obviously suggests that synthesis compounds based on the structure combination principle to discover more efficient and low toxicity anti-tumor compounds is of great research value.

## Methods

### Chemistry information

GA was purchased from State Uni-Bio Technology Co., Ltd., Baoji, China. Cinnamic acid and its derivatives were obtained from Aladdin Bio-Chem Technology Co., Ltd., China and Alfa Aesar Chemical Co., Ltd., Tianjin, China. The purity of all the materials, including GA, cinnamic acid and its derivatives are more than 98% by commercial purchase. Reagents of analytical reagent grade were provided by Beijing Chemical Plant. All reagents and chemicals were used as received without further purification unless specific stated. The aluminum sheets covered silica gel (Qingdao Haiyang Chemical Co., Qingdao, China) were used to monitor the reactions.

The melting points of purified products were obtained on an X-5 micro melting- point apparatus (Beijing Tech Instrument Co., Ltd., Beijing, China). ^1^H-NMR and ^13^C-NMR assays were recorded on a BRUKER AVANCE 500 or 400 NMR spectrometer (Fällanden, Switzerland). ESI–MS were recorded on a Thermo Scientific TM LTQ Orbitrap XL hybrid FTMS instrument (Thermo Technologies, New York, NY, USA). The final products were purified on 200–300 mesh silica gel. Cellular morphologies were observed using an inverted fluorescence microscope (Olympus IX71, Tokyo, Japan). On the last step reaction the yields can be calculated.

#### General procedure for the preparation of glycyrrhetinic acid–cinnamic acid hybrids 1a–9a

The corresponding cinnamic acid derivatives (6.0 mmol) and DMAP (0.6 mmol) were mixed in 50 mL dry DCM, as well as DCC. After dissolution, the glycyrrhetinic acid (3.5 mmol) was added. The mixture was stirred under nitrogen atmosphere at room temperature for 24 h. Afterwards, the solution was washed with brine, dried over sodium sulfate, filtered and concentrated. To yield pure product, the crude product was purified by flash chromatography (dichloromethane: methanol = 200:1).

*Cinnamoyl*-*3β*-*hydroxy*-*11*-*oxoolean*-*12*-*en*-*30*-*oic acid* (**1a**). White solid, yield: 45.0%, m.p.: 315.2–318.8 °C. ^1^H-NMR (500 MHz, CDCl_3_) (ppm): 0.85, 0.93, 0.97, 1.15, 1.20, 1.24, 1.39 (s, each, 3H, 7× –CH_3_), 4.67 (m, 1H), 5.73 (s, 1H, =CH–), 6.45 (d, *J* = 16.0 Hz, 1H, –CH=), 7.35–7.40 (m, 3H, Ar–H), 7.53–7.54 (m, 2H, Ar–H), 7.67 (d, *J* = 16.0 Hz, 1H, –CH=), 1.00–3.00 (22H, methyl- and methylene- of triterpenoid structure). ^13^C-NMR (125 MHz, CDCl_3_) (ppm): 16.6, 17.0, 17.5, 18.8, 23.5, 23.8, 26.6, 26.6, 28.3, 28.6, 28.7, 31.1, 32.0, 32.9, 37.1, 37.9, 38.5, 39.0, 41.0, 43.4, 43.9, 45.6, 48.4, 55.2, 61.9, 80.8, 119.0, 128.2, 128.6, 129.0, 130.3, 134.7, 144.5, 167.0, 169.6, 181.6 (–COOH), 200.5 (–C=O). MS (ESI) m/z: 599 [M-H]^−^, calcd. for C_39_H_52_O_5_ 600.

*(4*-*Methyl) cinnamoyl*-*3β*-*hydroxy*-*11*-*oxoolean*-*12*-*en*-*30*-*oic acid* (**2a**). White solid, yield: 47.3%, m.p.: 316.6–318.1 °C. ^1^H-NMR (500 MHz, CDCl_3_) (ppm): 0.84, 0.92, 0.96, 1.14, 1.19, 1.23, 1.38 (s, each, 3H, 7× –CH_3_), 2.37 (s, 3H, –CH_3_), 4.66 (m, 1H), 5.72 (s, 1H, =CH–), 6.40 (d, *J* = 16.0 Hz, 1H, –CH=), 7.18 (d, *J* = 8.0 Hz, 2H, Ar–H), 7.43 (d, *J* = 8.0 Hz, 2H, Ar–H), 7.64 (d, *J* = 16.0 Hz, 1H, –CH=), 1.00–3.00 (22H, methyl- and methylene- of triterpenoid structure). ^13^C-NMR (125 MHz, CDCl_3_) (ppm): 16.6, 17.0, 17.5, 18.8, 21.6, 23.5, 23.8, 26.5, 26.6, 28.2, 28.6, 28.7, 31.0, 32.0, 32.9, 37.1, 37.9, 38.4, 38.9, 41.0, 43.4, 43.9, 45.6, 48.4, 55.2, 61.9, 80.7, 117.8, 128.2, 128.6, 129.7, 131.9, 140.7, 144.5, 167.2, 169.6, 181.7 (–COOH), 200.5 (–C=O). MS (ESI) m/z: 613 [M-H]^−^, calcd. for C_40_H_54_O_5_ 614.

*(2*-*Methoxy) cinnamoyl*-*3β*-*hydroxy*-*11*-*oxoolean*-*12*-*en*-*30*-*oic acid* (**3a**). White solid, yield: 45.7%, m.p.: 317.0–319.2 °C. ^1^H-NMR (500 MHz, CDCl_3_) (ppm): 0.84, 0.92, 0.96, 1.14, 1.19, 1.23, 1.38 (s, each, 3H, 7× –CH_3_), 3.88 (s, 3H, –OCH_3_), 4.66 (m, 1H), 5.72 (s, 1H, =CH–), 6.52 (d, *J* = 16.0 Hz, 1H, –CH=), 6.91 (d, *J* = 8.0 Hz, 1H, Ar–H), 6.95 (t, *J* = 7.5 Hz, 1H, Ar–H), 7.35 (m, 1H, Ar–H), 7.51 (m, 1H, Ar–H), 7.98 (d, *J* = 16.0 Hz, 1H, –CH=), 1.00–3.00 (22H, methyl- and methylene- of triterpenoid structure). ^13^C-NMR (125 MHz, CDCl_3_) (ppm): 16.6, 17.0, 17.5, 18.8, 23.5, 23.8, 26.5, 26.6, 28.2, 28.6, 28.7, 31.0, 32.0, 32.8, 37.1, 37.8, 38.4, 38.9, 40.9, 43.3, 43.9, 45.6, 48.4, 55.1, 55.6 (–OCH3), 61.8, 80.5, 111.2, 119.4, 120.8, 123.6, 128.5, 129.0, 131.5, 139.9, 158.4, 167.5, 169.7, 181.7 (–COOH), 200.6 (–C=O). MS (ESI) m/z: 629 [M-H]^−^, calcd. for C_40_H_54_O_6_ 630.

*(3*-*Methoxy) cinnamoyl*-*3β*-*hydroxy*-*11*-*oxoolean*-*12*-*en*-*30*-*oic acid* (**4a**). White solid, yield: 65.5%, m.p.: 316.8–319.1 °C, ^1^H-NMR (400 MHz, CDCl_3_) (ppm): 0.84, 0.93, 0.97, 1.15, 1.20, 1.24, 1.39 (s, each, 3H, 7× –CH_3_), 3.84 (s, 3H, –OCH_3_), 4.67 (m, 1H), 5.72 (s, 1H, =CH–), 6.43 (d, *J* = 16.0 Hz, 1H, –CH=), 6.93 (dd, *J* = 8.0, 2.0 Hz, 1H, Ar–H), 7.05 (s, 1H, Ar–H), 7.13 (d, *J* = 8.0 Hz, 1H, Ar–H), 7.28–7.32 (m, 1H, Ar–H), 7.63 (d, *J* = 16.0 Hz, 1H, –CH=), 1.00–3.00 (22H, methyl- and methylene- of triterpenoid structure). ^13^C-NMR (100 MHz, CDCl_3_) (ppm): 16.6, 17.0, 17.6, 18.9, 23.5, 23.9, 26.6, 26.7, 28.3, 28.6, 28.7, 31.1, 32.0, 32.9, 37.2, 37.9, 38.5, 39.0, 41.1, 43.4, 44.0, 45.6, 48.4, 55.2, 55.5 (–OCH_3_), 61.9, 80.9, 113.0, 116.3, 119.3, 120.9, 128.7, 130.0, 136.1, 144.4, 160.1, 166.9, 169.5, 181.4 (–COOH), 200.4 (–C=O). MS (ESI) m/z: 629 [M-H]^−^, calcd. for C_40_H_54_O_6_ 630.

*(4*-*Methoxy) cinnamoyl*-*3β*-*hydroxy*-*11*-*oxoolean*-*12*-*en*-*30*-*oic acid* (**5a**). White solid, yield: 49.4%, m.p.: 316.9–319.1 °C. ^1^H-NMR (500 MHz, CDCl_3_) (ppm): 0.84, 0.92, 0.95, 1.14, 1.19, 1.23, 1.38 (s, each, 3H, 7× –CH_3_), 3.83 (s, 3H, –OCH_3_), 4.65 (m, 1H), 5.72 (s, 1H, =CH–), 6.32 (d, *J* = 16.0 Hz, 1H, –CH=), 6.90 (d, *J* = 8.5 Hz, 2H, Ar–H), 7.48 (d, *J* = 8.5 Hz, 2H, Ar–H), 7.62 (d, *J* = 16.0 Hz, 1H, –CH=), 1.00–3.00 (22H, methyl- and methylene- of triterpenoid structure). ^13^C-NMR (125 MHz, CDCl_3_) (ppm): 16.6, 17.0, 17.5, 18.8, 23.5, 23.8, 26.5, 26.6, 28.2, 28.6, 28.7, 31.0, 32.0, 32.8, 37.1, 37.8, 38.4, 38.9, 41.0, 43.3, 43.9, 45.6, 48.4, 55.2, 55.6 (–OCH_3_), 61.8, 80.5, 114.4, 116.4, 127.4, 128.6, 129.8, 144.1, 161.4, 167.3, 170.0, 181.6 (–COOH), 200.6 (–C=O). MS (ESI) m/z: 631 [M + H]^+^, calcd. for C_40_H_54_O_6_ 630.

*(2,3*-*Dimethoxy) cinnamoyl*-*3β*-*hydroxy*-*11*-*oxoolean*-*12*-*en*-*30*-*oic acid* (**6a**). White solid, yield: 53.4%, m.p.: 315.1–318.4 °C. ^1^H-NMR (400 MHz, CDCl_3_) (ppm): 0.84, 0.93, 0.96, 1.14, 1.20, 1.24, 1.39 (s, each, 3H, 7× –CH_3_), 3.86 (s, 3H, –OCH_3_), 3.88 (s, 3H, –OCH_3_), 4.66 (m, 1H), 5.72 (s, 1H, =CH–), 6.47 (d, *J* = 16.2 Hz, 1H, –CH=), 6.93 (dd, *J* = 8.0, 1.2 Hz, 1H, Ar–H), 7.05 (t, *J* = 8.0 Hz, 1H, Ar–H), 7.17 (dd, *J* = 8.0, 1.2 Hz, 1H, Ar–H), 8.00 (d, *J* = 16.0 Hz, 1H, –CH=), 1.00–3.00 (22H, methyl- and methylene- of triterpenoid structure). ^13^C-NMR (100 MHz, CDCl_3_) (ppm): 16.6, 17.0, 17.6, 18.9, 23.6, 23.9, 26.6, 26.7, 28.3, 28.6, 28.7, 31.1, 32.0, 32.9, 37.2, 37.9, 38.5, 39.0, 41.1, 43.4, 43.9, 45.7, 48.4, 55.3, 56.1 (–OCH_3_), 61.5 (–OCH_3_), 61.9, 80.8, 114.0, 119.4, 120.2, 124.3, 128.7, 128.9, 139.2, 148.6, 153.3, 167.1, 169.5, 181.3 (–COOH), 200.5 (–C=O). MS (ESI) m/z: 659 [M-H]^−^, calcd. for C_41_H_56_O_7_ 660.

*(2,5*-*Dimethoxy) cinnamoyl*-*3β*-*hydroxy*-*11*-*oxoolean*-*12*-*en*-*30*-*oic acid* (**7a**). White solid, yield: 57.1%, m.p.: 316.4–319.1 °C. ^1^H-NMR (400 MHz, CDCl_3_) (ppm): 0.85, 0.94, 0.98, 1.16, 1.21, 1.25, 1.40 (s, each, 3H, 7× –CH_3_), 3.80 (s, 3H, –OCH_3_), 3.85 (s, 3H, –OCH_3_), 4.68 (m, 1H), 5.72 (s, 1H, =CH–), 6.49 (d, *J* = 16.1 Hz, 1H, –CH=), 6.90 (m, 1H, Ar–H), 6.85 (brs, 1H, Ar–H), 7.06 (m, 1H, Ar–H), 7.96 (d, *J* = 16.1 Hz, 1H, –CH=), 1.00–3.00 (22H, methyl- and methylene- of triterpenoid structure). ^13^C-NMR (100 MHz, CDCl_3_) (ppm): 16.6, 17.0, 17.6, 18.9, 23.5, 23.9, 26.6, 26.7, 28.3, 28.6, 28.7, 31.1, 32.1, 32.9, 37.2, 37.9, 38.5, 39.0, 41.1, 43.4, 43.9, 45.7, 48.4, 55.3, 56.0 (–OCH_3_), 56.3 (–OCH_3_), 61.9, 80.7, 112.7, 113.6, 117.2, 119.6, 124.4, 128.7, 139.7, 153.0, 153.7, 167.3, 169.4, 181.0 (–COOH), 200.4 (–C=O). MS (ESI) m/z: 659 [M-H]^−^, calcd. for C_41_H_56_O_7_ 660.

*(2,3,4*-*Trimethoxy) cinnamoyl*-*3β*-*hydroxy*-*11*-*oxoolean*-*12*-*en*-*30*-*oic acid* (**8a**). White solid, yield: 66.7%, m.p.: 315.1–317.9 °C. ^1^H-NMR (400 MHz, CDCl_3_) (ppm): 0.84, 0.93, 0.96, 1.15, 1.20, 1.24, 1.39 (s, each, 3H, 7× –CH_3_), 3.87 (s, 3H, –OCH_3_), 3.89 (s, 3H, –OCH_3_), 3.92 (s, 3H, –OCH_3_), 4.66 (m, 1H), 5.72 (s, 1H, =CH–), 6.40 (d, *J* = 16.0 Hz, 1H, –CH=), 6.69 (d, *J* = 8.8 Hz, 1H, Ar–H), 7.28 (d, *J* = 8.8 Hz, 1H, Ar–H), 7.88 (d, *J* = 16.0 Hz, 1H, –CH=), 1.00–3.00 (22H, methyl- and methylene- of triterpenoid structure). ^13^C-NMR (100 MHz, CDCl_3_) (ppm): 16.6, 17.0, 17.6, 18.9, 23.5, 23.9, 26.6, 26.7, 28.3, 28.6, 28.7, 31.1, 32.0, 32.9, 37.2, 37.9, 38.5, 39.0, 41.1, 43.4, 44.0, 45.7, 48.4, 55.3, 56.2 (–OCH_3_), 61.0 (–OCH_3_), 61.6 (–OCH_3_), 61.9, 80.6, 107.8, 117.8, 121.8, 123.2, 128.7, 139.4, 142.6, 153.4, 155.6, 167.5, 169.4, 181.5 (–COOH), 200.4 (–C=O). MS (ESI) m/z: 689 [M-H]^−^, calcd. for C_42_H_58_O_8_ 690.

*(3,4,5*-*Trimethoxy) cinnamoyl*-*3β*-*hydroxy*-*11*-*oxoolean*-*12*-*en*-*30*-*oic acid* (**9a**). White solid, yield: 55.2%, m.p.: 315.3–317.7 °C. ^1^H-NMR (400 MHz, CDCl_3_) (ppm): 0.84, 0.93, 0.97, 1.15, 1.20, 1.24, 1.39 (s, each, 3H, 7× –CH_3_), 3.88 (s, 3H, –OCH_3_), 3.89 (s, 6H, 2× –OCH_3_), 4.68 (m, 1H), 5.72 (s, 1H, =CH–), 6.35 (d, *J* = 16.0 Hz, 1H, –CH=), 6.75 (s, 2H, Ar–H), 7.58 (d, *J* = 16.0 Hz, 1H, –CH=), 1.00–3.00 (22H, methyl- and methylene- of triterpenoid structure). ^13^C-NMR (100 MHz, CDCl_3_) (ppm): 16.6, 17.0, 17.5, 18.8, 23.5, 23.9, 26.5, 26.6, 28.3, 28.6, 28.7, 31.1, 32.0, 32.9, 37.1, 37.9, 38.5, 39.0, 41.0, 43.4, 43.9, 45.6, 48.4, 55.2, 56.3 (–OCH_3_), 61.1 (–OCH_3_), 61.9, 80.8, 105.3, 118.2, 128.6, 130.2, 140.1, 144.5, 153.6, 167.0, 169.7, 181.5 (–COOH), 200.5 (–C=O). MS (ESI) m/z: 689 [M-H]^−^, calcd. for C_42_H_58_O_8_ 690.

#### General procedure for the preparation of glycyrrhetinic acid–cinnamic acid hybrids 1b–4b

Compounds **1a**, **6a**, **8a**, **9a** (2 mmol) were hydrogenated by Pd/C (10%; 50.0 mg) in 30 mL THF. The mixture was stirred at room temperature for 2 h and filtered to remove Pd/C. The filtrate was concentrated in vacuum.

*Phenylpropanoyl*-*3β*-*hydroxy*-*11*-*oxoolean*-*12*-*en*-*30*-*oic acid* (**1b**). White solid, yield: 94.0%, m.p.: 313.6–316.5 °C. ^1^H-NMR (400 MHz, CDCl_3_) (ppm): 0.80, 0.84, 0.84, 1.13, 1.15, 1.23, 1.27 (s, each, 3H, 7× –CH_3_), 2.64 (m, 2H, –CH_2_), 2.96 (m, 2H, –CH_2_), 4.52 (m, 1H), 5.71 (s, 1H, =CH–), 7.17–7.22 (m, 3H, Ar–H), 7.27–7.30 (m, 2H, Ar–H), 1.00–3.00 (22H, methyl- and methylene- of triterpenoid structure). ^13^C-NMR (100 MHz, CDCl_3_) (ppm): 16.5, 16.7, 17.5, 18.8, 23.5, 23.7, 26.6, 26.6, 28.1, 28.6, 28.7, 31.1, 31.3, 32.0, 32.9, 36.4, 37.1, 37.9, 38.2, 38.9, 41.0, 43.4, 43.9, 45.6, 48.4, 55.2, 61.9, 80.9, 126.4, 128.4, 128.6, 140.7, 169.6, 172.9, 181.7 (–COOH), 200.5 (–C=O). MS (ESI) m/z: 601 [M-H]^−^, calcd. for C_39_H_54_O_5_ 602.

*(2,3*-*Dimethoxy) phenylpropanoyl*-*3β*-*hydroxy*-*11*-*oxoolean*-*12*-*en*-*30*-*oic acid* (**2b**). White solid, yield: 97.3%, m.p.: 317.4–319.1 °C. 1H-NMR (400 MHz, CDCl_3_) (ppm): 0.83, 0.83, 0.85, 1.13, 1.15, 1.23, 1.37 (s, each, 3H, 7× –CH_3_), 2.59–2.64 (m, 2H, –CH_2_), 2.93–2.98 (m, 2H, –CH_2_), 3.84 (s, 3H, –OCH_3_), 3.85 (s, 3H, –OCH_3_), 4.51 (m, 1H), 5.71 (s, 1H, =CH–), 6.80–6.77 (m, 2H, Ar–H), 6.97 (t, *J* = 8.0 Hz, 1H, Ar–H), 1.00–3.00 (22H, methyl- and methylene- of triterpenoid structure). ^13^C-NMR (100 MHz, CDCl_3_) (ppm): 16.5, 16.8, 17.5, 18.8, 23.5, 23.7, 25.7, 26.6, 26.6, 28.2, 28.6, 28.7, 31.1, 32.0, 32.9, 35.4, 37.1, 37.9, 38.2, 39.0, 41.0, 43.4, 43.9, 45.6, 48.4, 55.2, 55.9 (–OCH_3_), 60.7 (–OCH_3_), 61.8, 80.7, 110.9, 121.9, 124.0, 128.6, 134.5, 147.4, 152.9, 169.6, 173.1, 181.5 (–COOH), 200.5 (–C=O). MS (ESI) m/z: 661 [M-H]^−^, calcd. for C_41_H_58_O_7_ 662.

*(2,3,4*-*Trimethoxy) phenylpropanoyl*-*3β*-*hydroxy*-*11*-*oxoolean*-*12*-*en*-*30*-*oic acid* (**3b**). White solid, yield: 95.5%, m.p.: 316.3–319.1 °C. ^1^H-NMR (400 MHz, CDCl_3_) (ppm): 0.81, 0.83, 0.84, 1.12, 1.15, 1.22, 1.37 (s, each, 3H, 7× –CH_3_), 2.58 (t, *J* = 8.0 Hz, 2H, –CH_2_), 2.88 (t, *J* = 8.0 Hz, 2H, –CH_2_), 3.83 (s, 3H, –OCH_3_), 3.85 (s, 3H, –OCH_3_), 3.89 (s, 3H, –OCH_3_), 4.50 (m, 1H), 5.71 (s, 1H, =CH–), 6.57 (d, *J* = 8.8 Hz, 1H, Ar–H), 6.83 (d, *J* = 8.8 Hz, 1H, Ar–H), 1.00–3.00 (22H, methyl- and methylene- of triterpenoid structure). ^13^C-NMR (100 MHz, CDCl_3_) (ppm): 16.5, 16.8, 17.5, 18.8, 23.5, 23.7, 25.7, 26.5, 26.6, 28.1, 28.6, 28.7, 31.0, 32.0, 32.8, 35.6, 37.0, 37.8, 38.2, 38.9, 41.0, 43.3, 43.9, 45.6, 48.4, 55.1, 56.1 (–OCH_3_), 60.8 (–OCH_3_), 61.0 (–OCH_3_), 61.8, 80.6, 107.2, 123.9, 126.6, 128.5, 142.4, 152.0, 152.5, 169.6, 173.2, 182.0 (–COOH), 200.5 (–C=O). MS (ESI) m/z: 691 [M-H]^−^, calcd. for C_42_H_60_O_8_ 692.

*(3,4,5*-*Trimethoxy) phenylpropanoyl*-*3β*-*hydroxy*-*11*-*oxoolean*-*12*-*en*-*30*-*oic acid* (**4b**). White solid, yield: 95.5%, m.p.: 315.1–319.7 °C. ^1^H-NMR (400 MHz, CDCl_3_) (ppm): 0.79, 0.83, 0.85, 1.12, 1.15, 1.23, 1.37 (s, each, 3H, 7× –CH_3_), 2.63 (t, *J* = 8.0 Hz, 2H, –CH_2_), 2.90 (t, *J* = 8.0 Hz, 2H, –CH_2_), 3.81 (s, 3H, –OCH_3_), 3.84 (s, 6H, 2× –OCH_3_), 4.53 (m, 1H), 5.71 (s, 1H, =CH–), 6.42 (s, 2H, Ar–H), 1.00–3.00 (22H, methyl- and methylene- of triterpenoid structure). ^13^C-NMR (100 MHz, CDCl_3_) (ppm): 16.5, 16.9, 17.5, 18.8, 23.5, 23.8, 26.5, 26.6, 28.1, 28.6, 28.7, 31.1, 31.6, 32.0, 32.8, 36.5, 37.1, 37.9, 38.2, 38.9, 41.0, 43.4, 43.9, 45.6, 48.4, 55.2, 56.2 (–OCH_3_), 61.0 (–OCH_3_), 61.8, 80.9, 105.4, 128.6, 136.5, 136.6, 153.3, 169.6, 172.8, 181.6 (–COOH), 200.5 (–C=O). MS (ESI) m/z: 691 [M-H]^−^, calcd. for C_42_H_60_O_8_ 692.

### Bio-evaluation methods

#### Cell culture

HepG2 (human hepatocellular carcinoma), Hela (human cervical cancer), HT-29 (human colon carcinoma), MDCK (Madin–Darby canine kidney), H9C2 (rat myocardial cells) and HY926 were purchased from the Chinese Academy of Medical Sciences and Peking Union Medical College. All of these cell lines were maintained in RPMI-1640 (DMEM) supplemented with 10% (v/v) fetal bovine serum (FBS) and 1% (v/v) penicillin/streptomycin (Thermo Technologies, New York, NY, USA) under a humidified atmosphere containing 5% CO_2_ at 37 °C. To provide stock solutions which were used to prepare various concentrations of treatment media, GA and hybrids were added a volume of DMSO.

#### Cytotoxicity evaluation

The cytotoxicity of these compounds was evaluated on three human cancer cell lines (HepG2, HT-29, Hela) and three non-tumor cells lines (MDCK, HY926, H9C2) and MTT assay was performed to detect the cell proliferation. Simply, exponentially growing cells were cultured in 96-well plates (3 × 10^3^ cells/well). And the plates were placed in a humidified atmosphere for 72 h at 37 °C with 5% CO_2_. After added MTT solution (5 mg/mL) 20 μL to each well, the plate was incubated for a further 4 h. Then the cell supernatant was removed and 150 μL DMSO was added. Finally, the absorbance was determined at wavelength of 490 nm with the ELISA. Wells without drugs were used to be blanks. The IC_50_ values were calculated using Logit-method. The following Eq. (1) was proposed to calculate the inhibitory rate of cell growth:

1 $$\begin{aligned} \% {\text{ inhibition}} &= \left( {1 - {\text{Sample group OD}}} \right. \\ & \;\quad  \left. /{\text{Control group OD}} \right) \times 100\%\end{aligned} $$

#### Acute toxicity

Forty healthy Kunming mice (weight 18–22 g) of both sexes were purchased from Beijing Vital River Laboratory Animal Technology Company Limited (Beijing, China). The mice were divided into two groups matched the same weight and size, along with the standard in our previous studies [22]. In briefly, 20 mice were selected for blank control group, and the other 20 mice were for the treated group. All experiments followed the guidelines of “Regulation for the Administration of Affairs Concerning Experimental Animals” (State Council of China, 1988). All animals were placed in cages, fed with standard rodent chow and water ad libitum under a 12-h light–dark cycle. Before the experiments, the mice were deprived of food for 12 h but provided water freely. Then the treated group of both sexes were administered a single dose of **8a** (0.3 mL/10 g) via gavage twice a day, which was prepared in advance in bean oil solution with the maximum suspended dose (50 mg/mL). The total administration of **8a** was 3 g/kg during 8 h. And the blank control group were given bean oil by oral administration only. The mice’s general behavior was continuously noted in the first hour, intermittently observed in the next 4 h, and thereafter over a period of 24 h. Observations of the signs for toxicity and deaths were last for 14 days. The mortality response and behavior of toxic effects of mice were recorded.

#### Giemsa staining on Hela cells

Hela cells were seeded in 12-well plates (5 × 10^3^ cells/well), and then they were cultured for 24 h at 37 °C with 5% CO_2_. After that each group was added **8a**, making the final concentration of 0, 5, 10, 20 μM respectively. After 72 h, the cell supernatant was removed and the cells were cleaned with PBS twice, then kept in PBS/ethanol (1:1) for 2 min and fixed with cold ethanol for 10 min. After discarding ethanol, the cells were stained with Giemsa (Giemsa, Molecular Probes/Invitrogen Life Technologies, Carlsbad, CA, USA) stained for 5 min and washed with water. In the end, the cells were observed and photographed under inverted phase-contrast microscope at a magnification of 200×.

#### H33342 staining on Hela cells

H33342 staining was used to confirm whether the characteristic nuclear changes were associated with 8a in this assay. Hela cells were cultured in 12-well plates (5 × 10^4^ cells/well), which were placed in a humidified atmosphere for 24 h at 37 °C with 5% CO_2_. And then each group was added **8a**, making the final concentration of 0, 5, 10, 20 μM respectively. After 72 h, cell culture medium was discarded and the cells were washed twice with PBS. H33342 staining was then performed for 2 min and washed with water. In the end, the cells were observed and photographed under inverted phase-contrast microscope at a magnification of 200×.

#### Annexin V-FITC/propidium iodide (PI) assay

Hela cells were seeded in 12-well plates (5 × 10^4^ cells/well), which were placed in a humidified atmosphere for 24 h at 37 °C with 5% CO_2_. After treated with different concentrations of **8a** (5, 10, 20 μM) for 72 h, all cells were collected respectively with the right amount of trypsin (without EDTA) digestion. After added 1 mL cold PBS, the cells were gently suspended and centrifuged at 1000 rpm for 5 min. The harvested cells were suspended in 200 μL binding buffer which contained 10 μL Annexin V-FITC and PI. After avoided light reaction for 15 min, the cells were analyzed with a flow cytometry (BD, USA).

#### Caspase-3 assay

Hela cells were seeded in dishes at a density of 5 × 10^5^ cell per dish and the dishes were kept at 37 °C with 5% CO_2_ for 24 h. On the next day, various concentration of 8a (0, 5, 10, 20 μM) were added to the cells and cultured for 48 h. Pre-cooled PBS were added twice to wash the cells. After removal of the medium, ice-cold cell lysis buffer was added, and the cells were further incubated in an ice bath for 15 min, which contribute to the protein acquisition. The caspase-3 activity kit (Beyotime Institute of Biotechnology, Beijing, China) was used to determine the activity of caspase-3. A total of 50 μL cell lysates, 40 μL reaction buffer and 10 μL caspase-3 substrate (Ac-DEVDpNA) (2 mM) were added to 96-well plates. After 30 min of incubation, absorbance was measured at 405 nm, and samples were quantified with ELISA.
